# Mobile Health Technology Using a Wearable Sensorband for Female College Students With Problem Drinking: An Acceptability and Feasibility Study

**DOI:** 10.2196/mhealth.7399

**Published:** 2017-07-07

**Authors:** Noelle Regina Leonard, Michelle Silverman, Dawa Phuti Sherpa, Madeline A Naegle, Hyorim Kim, Donna L Coffman, Marcy Ferdschneider

**Affiliations:** ^1^ Center for Drug Use and HIV Research Rory Meyers College of Nursing New York University New York, NY United States; ^2^ Department of Health Behavior Studies Teachers College Columbia University New York, NY United States; ^3^ Rory Meyers College of Nursing New York University New York, NY United States; ^4^ University of Michigan Ann Arbor, MI United States; ^5^ Temple University Philadelphia, PA United States; ^6^ Medical Center Student Health Service Columbia University New York, NY United States

**Keywords:** wearable sensors, ecological momentary intervention, college students, alcohol use, feasibility studies, acceptability studies

## Abstract

**Background:**

An increasing number of mobile app interventions have been developed for problem drinking among college students; however, few studies have examined the integration of a mobile app with continuous physiological monitoring and alerting of affective states related to drinking behaviors.

**Objective:**

The aim of this paper was to evaluate the acceptability and feasibility of Mind the Moment (MtM), a theoretically based intervention for female college students with problem drinking that combines brief, in-person counseling with ecological momentary intervention (EMI) on a mobile app integrated with a wearable sensorband.

**Methods:**

We recruited 10 non-treatment seeking, female undergraduates from a university health clinic who scored a 3 or higher on the Alcohol Use Disorders Identification Test–Consumption (AUDIT-C) to participate in this pilot study. Study activities involved an in-person baseline intake and 1 follow-up assessment, 2 in-person alcohol brief intervention counseling sessions, and use of MtM technology components (sensorband and EMI on a mobile app) for approximately 3-4 weeks. The intervention used motivational interviewing (MI) and cognitive behavioral therapy (CBT) strategies for reducing risks associated with drinking. We used both qualitative and quantitative assessments to measure acceptability of the intervention and feasibility of delivery. Use patterns of the sensorband and mobile app were also collected.

**Results:**

Quantitative and qualitative data indicated high levels of acceptability for the MtM intervention. Altogether, participants made reports on the app on 26.7% (78/292) the days the technology was available to them and completed a total of 325 reports with wide variation between participants. Qualitative findings indicated that sensorband-elicited alerts promoted an increase in awareness of thoughts, feelings, and behaviors related to current environmental stressors and drinking behaviors in theoretically meaningful ways. Specific challenges related to functionality and form of the sensorband were identified.

**Conclusions:**

Delivering intervention material “just-in-time,” at the moment participants need to use behavioral strategies has great potential to individualize behavioral interventions for reducing problem drinking and other health behaviors. These findings provide initial evidence for the promise of wearable sensors for increasing potency of theoretically grounded mobile health interventions and point to directions for future research and uptake of these technologies.

## Introduction

Excessive consumption of alcohol by college students leads to numerous negative health, academic, and social consequences [1,2]. For college-age women in particular, problematic drinking is linked to unwanted sexual activity, assault, accident-related mortality and morbidity, poor academic performance, and interpersonal problems with friends or dating partners [3,4]. Brief, theoretically based, in-person interventions for problematic alcohol use among college students have demonstrated evidence of efficacy for reducing alcohol consumption and associated problems [5-7].

Increasingly, mobile phones have served as a platform to deliver brief interventions for problem drinking, typically in the form of text messages (short message service, SMS) [8-12]. These ecological momentary interventions (EMI) deliver reminders, prompts, or strategies to students in the real world [13] and typically are delivered at specific (eg, daily) intervals [9] or at times that coincide with drinking episodes, such as weekends or during university events [14-16]. Few, however, are informed by behavioral change theories [17]. Theoretically-grounded, blended interventions that provide support from both a real-life clinician and mobile technology have the potential to enhance motivation for behavior change [18-20].

### Increasing the Potency of Mobile Apps Through Wearable, Physiological Monitoring

The emergence of ambulatory monitoring of physiological markers of affective states allows users to receive real-time biofeedback through mobile phone apps. Wearable, noninvasive, unobtrusive technologies such as wristbands can be worn in daily life and have enormous potential to add potency to behavioral health interventions, including problematic alcohol use [9,21,22]. Electrodermal activity (EDA), also known as skin conductance, reflects changes in sympathetic nervous system activity (SNS) that is responsible for mobilizing the body to respond to emotional arousal such as stress and anxiety, including arousal associated with attention demanding tasks and arousal not open to conscious awareness. Alcohol-associated cues have been found to elicit SNS activity in individuals who are at risk for alcohol use disorders [23,24], particularly women [25]. When integrated with a mobile app on a smartphone, continuous, real-time monitoring of EDA can deliver EMI strategies when EDA increases, typically at the very moment individuals need to use behavioral health strategies in the course of their daily lives [21]. These interventions have been referred to as “just-in-time adaptive interventions,” where the support can occur at the time an individual is in a vulnerable state and highly susceptible to negative health outcomes [26]. Drawing individuals’ attention to these sensitive moments while they are occurring, and providing prompts or reminders of effective strategies, has great potential to avert immediate negative consequences and provide highly salient training experiences in using behavior change skills.

### Problematic Alcohol Use Among College-Age Women

Undergraduate students experience a significant developmental transition of increasing autonomy, marked by academic, personal, and social changes. Relative to males, female undergraduates consistently report experiencing a disproportionate amount of stress related to academic, social, financial, and other concerns [27]. Within this context, alcohol plays a conspicuous role on college campuses, as over 80% of college students in the United States report drinking alcohol [28]. While undergraduate females typically consume less alcohol than males, over the past 10 to 20 years, this gap has narrowed considerably, with a notable increase in the frequency and quantity of alcohol consumption and related problems among female undergraduate students [29]. For example, both males and females report similar rates of alcohol-induced blackouts [30]. Moreover, in addition to academic and interpersonal problems associated with excessive drinking, aggressiveness between dating partners and sexual violence increases while women are under the influence of alcohol [31,32].

Most college students report drinking for social reasons [33,34], although many students also report drinking to cope with negative emotional states [35-37]. The use of alcohol to manage negative affect including stress and anxiety, has been shown to be a risk factor for the development of alcohol use disorders [36-41], particularly in females [42-44]. Enhancing emotion regulation skills has been indicated as an important focus of interventions for problematic alcohol use [36,45].

### This Study

We describe a pilot intervention called Mind the Moment (MtM) for female college students with problematic drinking, using a blended intervention consisting of 2 brief, in-person sessions, along with the use of a mobile app and integrated sensorband for continuous measurement of EDA. Consistent with the extant models of brief alcohol interventions for college students, development of the MtM intervention was guided by cognitive behavioral therapy (CBT) and motivational interviewing (MI) strategies, which include providing personalized feedback on drinking patterns and motives for drinking, identifying triggers, establishing protective behavioral strategies, and planning to reduce drinking. Strategies to increase students’ emotional regulatory skills to deal with stress and triggers associated with excessive alcohol consumption may be particularly important for students who drink to cope with negative affect. These strategies include identifying current emotions, controlled breathing, mindfulness meditation, as well as individually-identified strategies such as listening to music or exercising.

Guided by the Unified Theory of Use and Acceptance of Technology (UTUAT) [46], an empirically derived grand theory of perceived advantages and challenges associated with new technologies, we explore the acceptability and feasibility of the MtM intervention components using quantitative and qualitative methods. While the UTAUT is designed for the work-place, it includes theories traditionally used to explain individual and social factors involved in behavioral health change. Additionally, we examined students’ patterns of use of the technology components and their perceptions of how the intervention addressed the theoretical targets of the intervention.

## Methods

### Participants and Procedures

**Figure 1:**
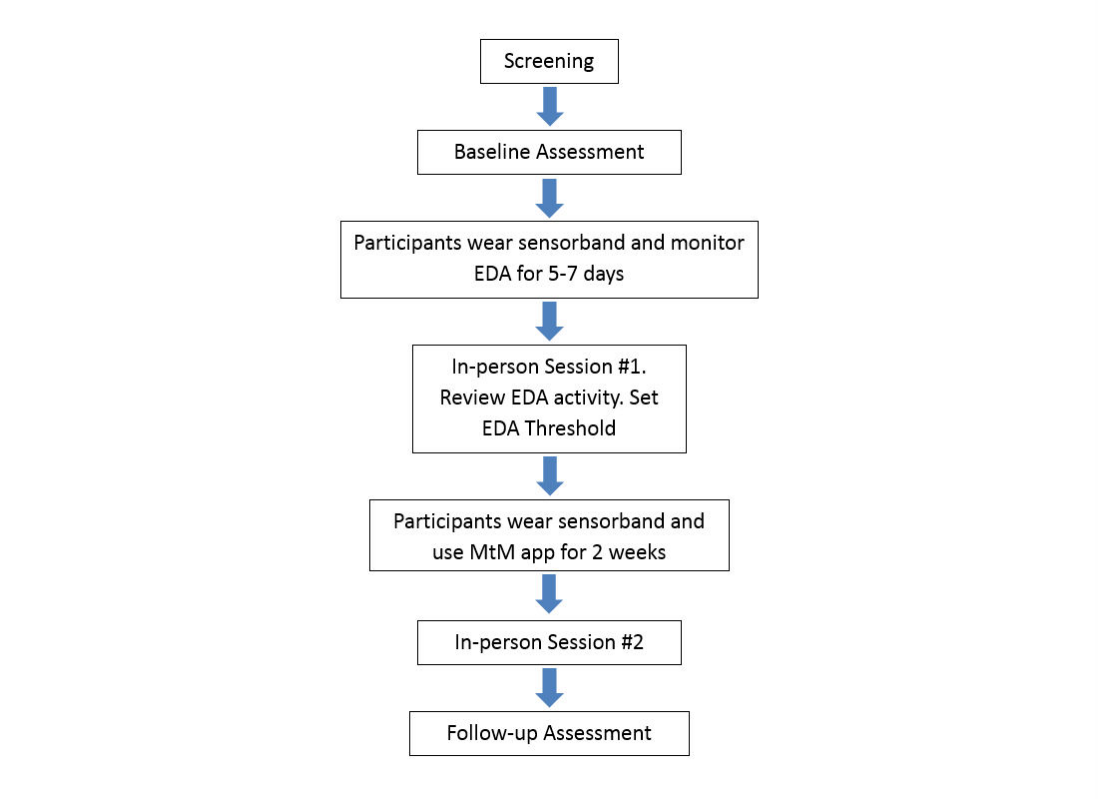
Study flow.

Participants (N=10) were nontreatment seeking undergraduate female students receiving routine medical care at the university health center (UHC) in a large, highly competitive private university that screens all patients for risky drinking. Female undergraduate students who score at or above the threshold for risky drinking for women (3 or higher) on the Alcohol Use Disorder Identification Test-Consumption (AUDIT-C) [5,47] are routinely provided with information by the UHC provider regarding what their score suggests and asked if they would like a referral to counseling about the potential for problems related to their alcohol use. Patients who agreed to receive a referral for counseling were provided with a brief description of the study by the UHC provider, and if interested, signed informed consent to be contacted by the research staff to receive more information about the study. Students who were not interested in the study received the UHC standard counseling referrals. Interested students were then screened by phone for eligibility by the research assistant (RA) using the following criteria: female (by birth), age 18-24 years, and approved by their health center provider for participation. Students were ineligible for participation if they were pregnant or currently receiving treatment for addiction or severe mental illness. After determining eligibility, the RA explained the study procedures: a baseline and follow-up assessment, 2 in-person counseling sessions concerning their alcohol use, and use of the MtM technology for approximately 3 weeks (Figure 1). Students who met eligibility criteria and were interested in participating were scheduled for a baseline assessment where they signed informed consent. Participants received US $25 for each assessment, $15 for each in-person counseling session, $25 per week for wearing the sensorband 5 or more hours a day (for 5 out of 7 days), and $5 for sending their data electronically every other day. All procedures were approved by the university’s institutional review board.

### Intervention Components

The intervention consisted of 2 main integrated components (Figure 1): the technology (MtM app, sensorband, and study smartphone) and 2 brief in-person counseling sessions. Participants’ use of the MtM technology took place in two phases; the first phase occurred between the baseline assessment and the first in-person session, and the second phase occurred between the first in-person session and the second in-person session or follow-up interview. Both the in-person and the MtM technology intervention components were developed for the study with input from members of the target population and guided by MI and CBT strategies [48-51]. This input included elements of the content of the app, such as typical emotions and situations experienced by students as well as protective behavioral strategies for reducing harms related to drinking and ways of reducing stress. Table 1 presents the theoretically based components of the intervention, including the mode of delivery (in-person or technology). Each in-person session lasted approximately 1 h and was conducted by an advanced practice nurse certified in mental health and addictions.

**Table 1:** Theory table.

Theory	Technique	Intervention component	Delivery method	Example
**MI^a^**	Elicit recognition of problem(s)	Drinking feedback	IS^b^	What are your thoughts about your AUDIT-C^c^ score? Do you think it places you at risk for health problems?
**CBT^d^, MI**	Assess risk	Risk self-assessment	IS	Would you say you sometimes are a “risky drinker?”
CBT, MI	Drinking context	Identify triggers or cues	IS, tech^e^	Do certain situations act as cues to drink?
MI	Motivation	Decisional balance	IS	Are there harms of drinking that you would like to avoid?
MI	Assess readiness	Readiness for change assessment	IS	On a scale of 1-10, how ready would you say you are to cut back or quit drinking?
CBT, MI	Goal-setting	Drinking plan	IS	If you choose to cut back, what would you want to consider in making your plan (eg, number of drinks and frequency)?
CBT	Identify feelings	Feeling scale	Tech	How are you feeling?
CBT	Coping	Coping statements and relaxation	Tech	Freeze, breathe, choose Guided meditation
CBT	Positive self-talk	Cool thoughts	Tech	My thoughts are not me
CBT	Protective behavioral strategies	Coping with triggers	IS, tech	Take a cab home

^a^MI: motivational interviewing.

^b^IS: in-person session.

^c^AUDIT-C: Alcohol Use Disorders Identification Test-Consumption.

^d^CBT: cognitive behavioral therapy.

^e^tech= Mind the Moment (MtM) technology.

### Mind the Moment Ecological Momentary Intervention

#### Instrumentation

**Figure 2:**
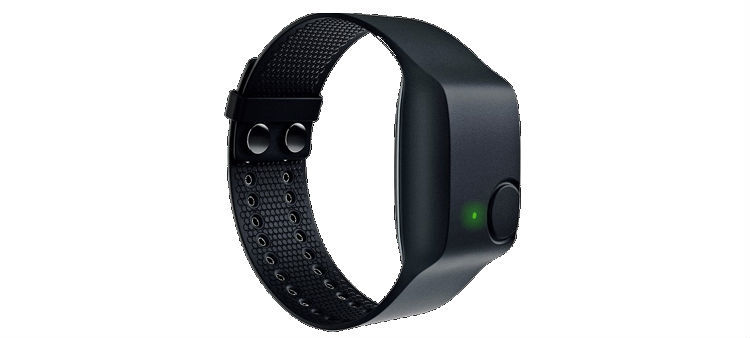
Empatica E4 sensorband.

The Empatica E4 [52] wearable wristband (“sensorband”) (Figure 2) measures EDA by applying constant low voltage to the skin and measuring the resultant current. It also contains an accelerometer and temperature sensor that informs interpretation of the EDA signal, which can depend on physical movement and changes in body temperature. The sensorband connects wirelessly to an Android-based smartphone via Bluetooth [53], and through the Empatica app, displays real-time EDA, heart rate, and temperature. The separate app for the MtM intervention was built on the Empatica application programming interface (API) and customized for the study using the Studio IDE [54] platform with open-source libraries support pulled from Github [55].

#### Implementation of Mind the Moment (MtM) Technology

After completing the baseline interview, participants were provided with a sensorband, a study Android smartphone, and instructions for use (Figure 1). In the first study phase, and before the first in-person session, participants were asked to wear the sensorband and carry the study phone as frequently as possible for 4 to 6 days, periodically checking their EDA using the Empatica app and making note of when they were aroused or stressed.

In the second phase, participants were provided with the MtM app on the study phone immediately after the first in-person session. In order to set the individual EDA threshold, together, the RA and participant reviewed her EDA and activities from the previous week and compared it with her baseline assessment activity to determine peaks due to physical exertion (eg, exercising and running to class) and mental or emotional stress (eg, taking an exam or receiving a grade). The RA then set the threshold to alert (vibrate or ring) the participant when her EDA rose above an individually determined threshold. Participants were provided with instructions for using the MtM app when alerted, and they could choose to respond, pause the alert for 10 min, or ignore it.

In addition to EDA-triggered reports, participants were encouraged to self-initiate MtM at any time. After the first 2 days of using the technology, each participant was contacted by the RA who inquired about her use of the technology and adjusted the threshold in the event that there were too many or too few alerts based on sensorband use and feelings of arousal.

#### Contents of the MtM App

**Figure 3:**
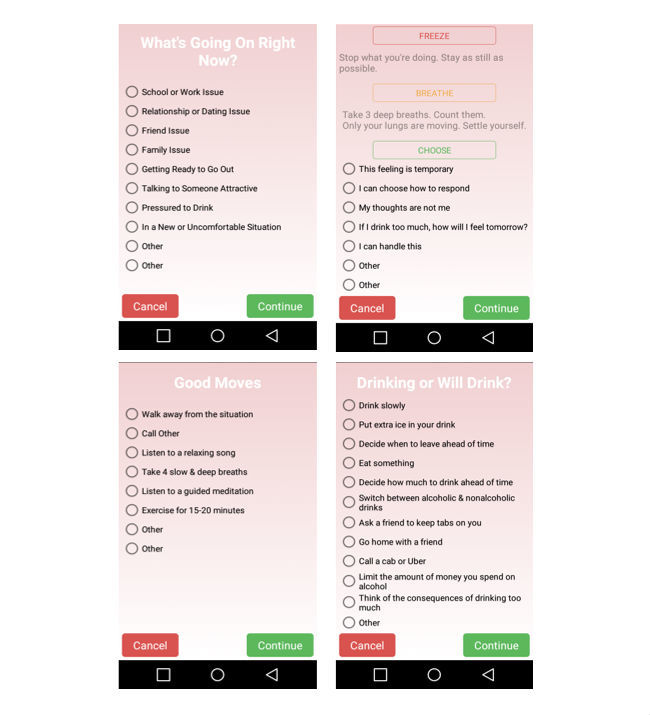
MtM app contents.

As seen in Figure 3, participants were led through a series of CBT-informed questions and strategies when they were alerted that their EDA reached threshold or when they made a self-initiated report. The first questions asked participants to identify their current emotions and level of intensity, and current context. When participants identified a positive emotion, the app supplied an affirmational statement and then closed. When a negative emotion was selected, participants were asked about their current context, provided with a brief CBT strategy, and asked about their intentions to drink alcohol. Based on their responses, specific coping strategies including a meditation, listening to a favorite song, positive self-talk, and protective behavioral strategies for drinking were offered. Participants were also encouraged to personalize the app by inserting specific emotions and contexts that were not on the preprogrammed list. These individually selected emotion and contexts then appeared whenever they responded to a sensor-triggered alert or when making a self-initiated report. Programming constraints did not make it possible to display both real-time EDA data using the Empatica app and the MtM app at the same time.

### In-Person Sessions

The first in-person session with the clinician was guided by the Southeastern Consortium for Substance Abuse Training (SECSAT) [56], a semistructured interview guide that includes assessing readiness, reviewing drinking patterns and cues, discussing harms or consequences related to drinking, eliciting protective behavior strategies, and developing short-term goals for reducing risky drinking. At the end of the session, the RA returned and demonstrated how to use the sensorband in conjunction with the MtM app. Participants were encouraged to wear the band and carry the study phone as often as possible during the next few weeks, particularly when they planned to drink.

The second in-person session with the clinician occurred approximately 3 weeks after the first session. During this session, the clinician discussed participant’s use of MtM app and sensorband in relation to triggers for drinking and their progress with drinking-related goals. Participants were asked to identify continuing barriers and facilitators of adhering to their drinking-related goals, plans for overcoming these barriers, and enhancing the facilitators. At the completion of the study, referrals were given to participants when the clinician believed they would benefit from further support.

### Assessments and Measures

#### Physiological Measures

##### Baseline Assessment of Electrodermal Activity (EDA)

In order to determine the individual EDA threshold for each participant, an assessment of baseline EDA was conducted to determine the full spectrum of EDA using a startle task, a cognitive stress task, and physical activity. At the beginning, participants were asked to sit quietly in a comfortable position for a few moments and after signing informed consent, the RA placed the Empatica sensorband on the participant’s right wrist and asked her to continue sitting quietly for a few moments. To elicit a startle reflex, a loud air horn, out of sight from the participant, was blown once. Participants also completed a computerized Stroop color word task in which they were asked to read the names of the colors as quickly as possible in 1 min, and finally, a brief physical task (eg, jumping jacks) was performed for approximately 4 min.

##### Alcohol Consumption

The Time Line Follow Back (TLFB) is a self-report measure of participants’ past 30-day alcohol use [57]. At the baseline assessment, participants were asked to indicate the number of drinks consumed each day over the past month using a calendar. We calculated the total number of drinking days and total number of drinks consumed over the past 30 days.

#### Acceptability and Feasibility Measures

##### Satisfaction With Intervention

We quantitatively assessed participants’ satisfaction with the MtM intervention using 5 items from the Client Satisfaction Questionnaire (CSQ) [58] and 6 additional items developed for the study. All items were rated on a 4-point scale. The CSQ items measured satisfaction with the intervention as a whole (eg, “How would you rate the quality of the service you received?”), whereas the other items assessed individual aspects of the technology intervention (eg, “How effective were the smartphone and sensorband in helping you meet your goals?”). The CSQ has been used with similar nontreatment seeking samples measuring satisfaction with mobile health interventions [59,60]. Higher scores indicate greater satisfaction. In addition, 6 open-ended questions regarding participants’ general satisfaction and feedback of the technology components, as well as barriers and facilitators of participants’ use of the technology were developed for the study. All questions were administered on a laptop computer with headphones using audio assisted interviewing of the Questionnaire Development Software [61].

##### Qualitative Interview

Immediately after the second in-person session with the clinician, students participated in a brief, (<30 min) semistructured qualitative interview with the clinician, where responses were recorded on paper. The purpose was to explore participants’ perceptions of the technology, including the challenges, and the barriers and facilitators of using it in their daily life to help them achieve their goals.

##### Usage Data

Data from the smartphone was collected, including the frequency with which participant’s reached their EDA threshold, the number and content of reports made on the app (sensorband-triggered and self-initiated), and the amount of time spent on each screen.

#### Qualitative Data Management and Analysis

Immediately after each follow-up interview, the interviewer reviewed the responses with the participant for accuracy. The qualitative interview responses and notes from the second in-person session was entered into Dedoose (Los Angeles, CA) [62]. Research team members created a “start list” [63] of initial codes based on the UTAUT model and the theoretical models of behavior change (MI and CBT). Codes consisted of labels or tags containing one to several words assigned to sections of the text that described that code. Guided by Grounded Theory [64], the research team then met to review the codes, develop the codebook, apply the start list codes to the text, and create new codes based on emergent themes.

## Results

### Participants

The UHC referred 58 female patients with AUDIT-C scores of 3 or higher and who signed informed consent allowing contact from the research staff. Patients were contacted by the research staff in the order the referrals were received until the target enrollment was met. Potential participants were contacted by an email, SMS text messaging, or phone call (depending on stated preference). Forty (71%) did not respond, and of the 18 who responded, 2 were no longer interested in the study and 2 were ineligible, leaving 14 eligible for participation. Overall, 11 participants enrolled, and 1 dropped out after the baseline assessment citing time constraints. Thus, 10 participants completed the baseline and follow-up assessments, participated in both in-person sessions, and used the MtM technology to varying degrees as detailed below.

The mean age of the participants was 20.7 (range=19-22). Four were seniors, 3 were juniors, 2 were sophomores, and 1 was a freshman. Participants were roughly representative of the main racial or ethnic groups of the university; 7 were white, 2 were Asian, and 1 was black. AUDIT-C scores ranged from 3 to 7 (mode=3). On the TLFB, the mean number of days drinking over the past 30 days was 12.11 (standard deviation [SD]=4.25, range=7-30 days), and the mean number of drinks consumed was 46.66 (SD=33.73, range=8-127 drinks). The mean number of days that elapsed between the baseline and follow-up assessments was 39.7 days (SD 12.92).

### Feasibility, Acceptability, and Usage

On the quantitative measure of acceptability (Table 2), the overall average rating on the 5 CSQ items measuring satisfaction with the intervention was 3.4 on the 4-point scale. All participants reported that the intervention helped them deal more effectively with their problems and that they met at least some of their personal goals in reducing risky drinking. All participants found the technology component “somewhat” or “very easy” to learn, although there was significant variability in the level of satisfaction with the sensorband and the MtM app. Specifically, 60% (6/10) of participants were “mostly” or “very” satisfied with the sensorband and 50% (5/10) with the app. Seventy percent (7/10) of the participants noted that the MtM intervention was “somewhat” or “very” effective in helping them reduce their risky drinking.

**Table 2:** Acceptability Questionnaire (n=10).

Survey item	Mean (SD^a^)
How would you rate the quality of service you received?^b^	3.60 (0.70)
To what extent has our program met your needs?^b^	3.50 (0.71)
If a friend were in need of similar help, would you recommend our program to him or her?^b^	3.20 (0.42)
How satisfied are you with the amount of help you received?^b^	3.50 (0.71)
Have the services you received helped you deal more effectively with your problems?^b^	3.00 (0.00)
To what extent have you met your personal goals in reducing risky drinking?^c^	2.60 (0.70
How satisfied are you with the sensorband?^c^	2.60 (0.84)
How satisfied are you with the smartphone app?^c^	2.60 (0.70)
How effective were the smartphone app and sensorband in helping you meet your goal?^c^	2.30 (0.95)
How would you rate your experience learning to use the sensorband and smartphone app?^c^	3.60 (0.52)
How does this experience compare with other times you have tried to reduce risky drinking?^c^	3.00 (1.05)

^a^SD: standard deviation.

^b^Items are from the Client Satisfaction Questionnaire [46].

^c^Items were developed for the study. All scores were based on a 4-point Likert scale with higher scores indicating more acceptability.

After adjusting the EDA thresholds and the sensorband connection for most participants during the first few days of using the sensorband and MtM app, all participants reported that the sensorband alerts they received were usually valid. That is, they were typically emotionally aroused when they received the alert that their EDA had reached the threshold and were aware that physical activity had the potential to trigger an alert. The number of times participants rated each emotion is displayed in rank order in Table 3. “Stress” (68 occurrences) was the most frequently reported emotion while “anxiety” and “fatigue” tied for the second most reported emotion (41 occurrences each). The more positively valenced emotions of “satisfied” (28 occurrences) and “excited or energized” (26 responses) were ranked 3^rd^ and 4^th^, reflecting the wide range of emotions associated with SNS arousal. As seen in Table 4, school or work issues constituted the overwhelming number of contexts reported by participants in response to sensor-triggers. The second most frequently chosen context was “other,” reflecting participants’ individually chosen specific contexts (eg, the name of a particular person or place).

**Table 3:** Rank ordered emotions for sensorband reports.

Emotion	# of reports
Stressed	68
Anxious or nervous	41
Tired	41
Satisfied	28
Excited or energized	26
Frustrated	15
Happy	9
Relieved	4
Sad	4
Embarrassed	1

**Table 4:** Rank ordered contexts for sensorband reports.

Context	# of reports
School or work issue	118
Other	23
Friend issue	13
Relationship or dating issue	7
Getting ready to go out	4
Talking to someone attractive	2
Family issue	2
In a new or uncomfortable situation	1

**Table 5:** Negative valance reports with screen time (seconds).

PID^a^	Total			Self				Sensor		
	# of reports	Sum	Mean (SD^b^)	# of reports	Sum	Mean (SD)	# of reports		Sum	Mean (SD)
303	15	576.03	38.40 (26.41)	1	91.27	91.27 (N/A^c^)	14		484.76	34.63 (22.82)
308	11	572.40	52.04 (31.03)	5	362.04	72.41 (30.21)	6		210.36	35.06 (20.84)
310	5	255.92	51.18 (28.12)	4	218.91	54.73 (31.16)	1		37.02	37.02 (N/A)
311	40	1057.55	26.44 (15.17)	1	20.64	20.64 (N/A)	39		1036.91	26.59 (15.34)
312	10	622.47	62.25 (30.10)	1	121.48	121.48 (N/A)	9		500.99	55.67 (23.07)
313	80	1512.66	18.91 (12.48)	4	203.51	50.88 (25.74)	76		1309.15	1723 (8.96)
314	5	373.20	74.64 (56.93)	1	171.04	171.04 (N/A)	4		202.16	50.54 (21.21)
315	19	1365.45	71.87 (67.08)	12	1199.63	99.97 (70.66)	7		165.82	23.69 (7.43)
316	11	401.64	36.51 (28.81)	0	0.00	0.00 (N/A)	11		401.64	36.51 (28.81)
317	9	499.40	55.49 (44.42)	6	328.44	54.74 (49.08)	3		170.95	56.98 (43.20)

PID: Participant ID

SD: standard deviation.

N/A: not applicable.

### Use Patterns

The number of days participants used the MtM app and sensorband ranged from 5 to 14 days. Altogether, participants made reports on 78 out of a total 292 days (26.71%) that the technology was available, with significant variation in their usage patterns. There was no statistically significant difference between the average amount of time participants wore the sensorband during the daytime hours (mean=29.32, SD=15.82) versus the evening hours (mean=24.17, SD=25.76).

**Figure 4:**
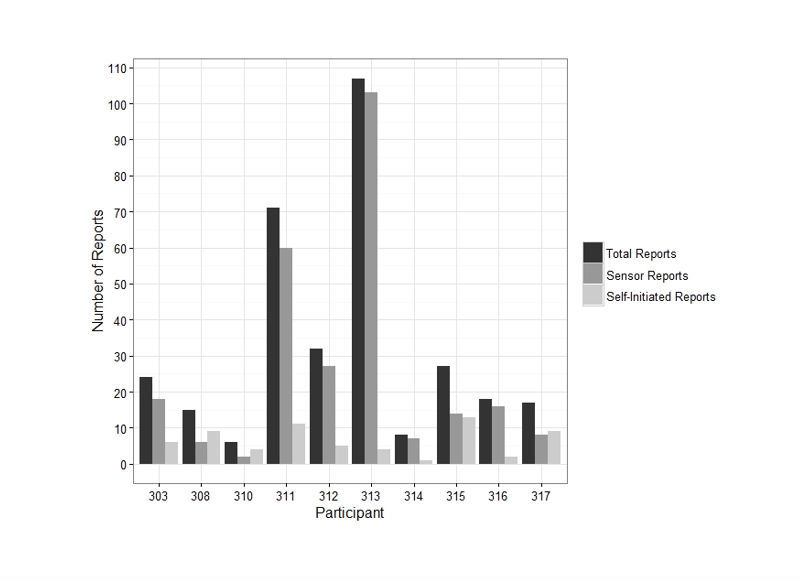
Number of completed reports by participants for each type of response (sensorband or self-initiated). The number of reports includes positive and negatively valenced emotions and personalized emotions (“other”).

Overall, participants completed a total of 325 reports (mean=32.5, SD=31.96), which included 261 sensorband triggered (mean=26.1, SD=31.7) and 64 (mean=6.4, SD=3.9) self-initiated reports. Figure 4 shows the number of total sensor-triggered and self-initiated reports for each participant. In light of the large number of sensor-trigged reports by participants 311 and 313, we examined the activity in each report for accuracy in reporting.

We calculated the total amount of time participants spent using the app by summing the amount of time they spent on each screen. On average, participants spent 17.50 min (SD 9.37) using the app, either in response to sensorband prompts or making a self-report. Reports that were not completed, that is, when participants did not get to the final screen, were not included.

By design, reporting a negative emotion generated a greater number of app screens for sensor-initiated and self-initiated reports. Thus, in Table 5, we examined the total number of negative emotion reports and the average amount of time participants spent on the app when responding to a sensor-initiated or self-initiated report. With the exception of P315 who made 12 self-reports, most participants completed less than 6 (total=35), in contrast with the larger number of sensor-initiated negative emotion reports (total=170).

### Qualitative Findings

#### Perceived Advantages of MtM

##### Prompting Awareness of Current Feelings

The predominant theme that emerged from the qualitative data focused on how MtM helped students access and identify their feelings and the intensity of those feelings. With one exception, all participants expressed that being notified of an increase in stress and responding to sensorband alerts through the app made them “think,” “be aware of,” “reflect,” “identify,” or “gauge” feelings and emotional states. This sentiment was expressed by participants regardless of the type of report and frequency of use. For example, as seen in Table 4, P316 received 15 sensorband alerts, whereas P317 received only 4. However, both participants remarked that MtM compelled them to reflect on their feelings. P317 noted, “I liked being forced to step back and assess my emotions,” whereas P316 indicated, “it allowed me to check in with myself and forced me to be mindful about my behavior and feelings.”

P314 received 7 sensorband alerts and initiated 1 self-report. She reported, “the band messages were helpful first when I was drinking and kind of depressed. It helped to put in what I was feeling.” P303 noted that, “It made me realize how my body reacts to mood change.” Others noted that the technology assisted them in coping with their feelings in situations not necessarily related to drinking. P308 found that, “the band reminds me that I can handle this, I can cope.” Moreover, she said that, “the band made me less anxious about tests.”

Finally, using the technology, particularly the messages on the app, made several participants feel emotionally supported. “...it was good to have the app to soothe me,” noted P317.

##### Increased Awareness of Alcohol Use

Of the 10 participants, 9 reported that one or both components of the intervention increased their awareness of their drinking behaviors to varying degrees.

For many, the technology components were instrumental in helping them adhere to the alcohol risk reduction plan they formulated during the first in-person session with the clinician. P303, for example, rated the sensorband and app as, “somewhat dissatisfying” on the quantitative acceptability measure. However, in her qualitative interview, she expressed that simply wearing the band was a reminder that, “I was on the plan.” She also noted that the screen which asked, “Drinking or Planning to Drink?” was a “really useful prompt and raised a question for me about drinking and reminded me of the options that I had.” Similarly, while P313 received the overwhelming majority of sensorband alerts and was “mostly satisfied” with the sensorband in her quantitative rating, she also indicated that, “the notifications of increased stress levels” helped her to engage in “reflective thinking” about what was causing her stress and how it related to her drinking, noting that it “helped me follow my plan to only drink once a week.”

For several participants, wearing the sensorband seemed to increase their consciousness of the amount of alcohol they were consuming. For example, P316 recounted that during the first phase of using the sensorband with the Empatica app, “I got a cider and watched the EDA number go up. Seeing an immediate congruence was really helpful.” She went on to explain that when using the MtM app, “Drinking with the band on was different...it made me think about the amount. I was surprised...”

Several participants recounted that the sensorband and MtM app helped them use protective behavioral strategies for alcohol use such as, “it restricted my purchases (of alcohol)” and “I decided not to go to a party” (P316). P310 found that, “I was able to drinker slower when using the band and slowing down worked well (to stick to my plan).”

For a minority of participants (3/10), the alerts sometimes increased their anxiety or level of stress. As described above, P303 felt that the alerts had positive effects, but also indicated that, “While I am dealing with stress I do not have to be reminded of the stress.”

### Perceived Challenges Associated With the MtM Intervention

#### Form and Functionality Issues

##### Issues Related to Form

Two primary issues related to the form of the band emerged in the qualitative data. Approximately half of the participants indicated that the band was “uncomfortable,” “bulky,” or “too large.” As a result, several participants felt “self-conscious” or “awkward” when wearing it, particularly if someone commented on it. Two participants indicated that using the technology in class was challenging. For example, P310 noted that while in class, “my professor commented on it which made me feel awkward,” whereas a few other participants related that they would not wear the band “with a nice outfit” (P312) or “going out with friends” (P308).

##### Charging Issues

The main complaint raised by the vast majority of participants was that they kept forgetting to charge the phone and sensorband. This problem mainly stemmed from the fact that for most participants, the study phone was not their main personal phone. “The phone and band were too cumbersome to carry around,” P313 recounted. Several participants related ways that they attempted to keep the study phone and band charged (eg, “I kept it near my daily jewelry”).

##### Connectivity Issues

Many participants indicated that the band and phone disconnected frequently, which was “a deterrent to using” as explained by P310. P317 noted that, “it was hard to tell when it was or was not connected.” Many participants understood that the connection problems were related to the Bluetooth technology that required constant proximity between the phone and the band. Several participants suggested that an alert be sent when the sensorband is not connected.

##### Inopportune Alerts and Attention

P314 indicated that, “it was annoying when it went off during class,” and other participants mentioned feeling self-conscious needing to explain the sensorband and their participation in the study when questioned by peers and professors.

## Discussion

### Principal Findings

We found a high level of acceptability for a wearable sensorband integrated with an EMI on a smartphone app for ambulatory physiological monitoring and alerting of heightened emotion among college-age women with drinking problems. Combined with 2 in-person, brief-counseling sessions, this blended intervention was feasible to deliver and demonstrates promise for individualizing mobile health (mHealth) interventions and adding potency to provider-delivered health and clinical interventions. Importantly, quantitative and qualitative data indicated that overall, participants found that the sensorband alerts were valid indicators of heightened emotion. For many, the alerts promoted an increase in their awareness of thoughts, feelings, and behaviors related to their environmental stressors and drinking behaviors, consistent with the theoretical model of the intervention and with other studies of EMIs [65]. To our knowledge, this is the first intervention developed using wearable mHealth technology for addressing drinking problems among college-age women.

Participants demonstrated a high degree of engagement with the technology regarding the number of days using the sensorband and app, the number of sensorband triggered reports, and the overall amount of time spent on the app. As expected, there was significant variability in the number of sensor-triggered reports made by participants. While sensor-triggered reports constituted the vast majority of responses on the app, all participants engaged in at least one self-report, indicating that participants seem to feel that the app was helpful.

### Barriers to Use of the Technology

As is typical with new technologies, participants encountered several challenges to consistent use of the technology components. For some, determining their individual EDA threshold required adjustment over the course of the first few days, but once adjusted, most participants continued to utilize the sensorband and the app throughout the duration of this brief pilot. In future research, we will examine more nuanced strategies for assessing and determining individuals’ EDA threshold. The primary barrier participants encountered involved carrying 2 phones (study phone and personal phone), resulting in participants forgetting to use the MtM technology, as well as frequent loss of the Bluetooth connection due to the distance between the study phone and the sensorband. Since the Empatica sensorband can also be used with iPhones, in future studies we will be able to program and install the MtM app on participants’ personal phones. Participants also provided instructive feedback regarding reducing the bulkiness of the sensorband and developing simple ways of turning off the alerts in particular contexts or at particular times. Future refinement of the timing of alerts is vital so that support is congruent with moments of need rather than at inopportune periods of time or when individuals are not experiencing the internal or contextual challenges that are the target of intervention [26]. For example, context sensing programs using global positioning systems (GPS) [66] or inputting students’ class schedules for example, can be incorporated into the app in order to prevent alerting in specific environments. Additionally, as wearable devices have become more mainstream, incorporating the measurement of EDA and other physiological markers of emotion into these devices may reduce participant burden, and increase their utilization and scalability.

### Emotions and Related Contexts Elicited by the Technology

Issues related to school or work emerged as the predominant context reported by our college student participants in response to sensor-triggered alerts. Consistent with the literature [67], within these contexts, stress, anxiety, and fatigue constituted the primary feelings associated with these challenging situations. Surprisingly, “tired” emerged as a frequently endorsed emotion associated with increase in participants’ EDA. Placing “tired” on the app was suggested by students in the target population in our development of the app, and we assumed that this emotion would be chosen by students while self-reporting rather than in response to increases in EDA. While in future research we will explore this emotional label in more depth, our qualitative interviews suggest that participants’ ability to cope with stressful academic or other situations was dampened by their level of fatigue, thus endorsing “tired” felt as if it was an appropriate choice.

We also noted that there was little variability in the responses of emotions by participants, as stress, anxiety, and fatigue were the most frequently reported feelings on the MtM app. The serial positions of stress and anxiety as first and second on the first screen of the MtM app may have influenced this reporting, thus in future research, we will explore other ways of presenting choices such as the circumplex model [54] or allowing participants to write in their specific emotion, which may limit this potential bias.

### Theoretically Based Behavior Change

Our qualitative findings indicated that the use of CBT strategies in participants’ real-world contexts promoted reflection of the type and nature of their individual stressors and the strategies they use to deal with them, including drinking. Although in this brief feasibility pilot we were unable to determine any intervention effects on drinking behaviors, these findings provide evidence that participants successfully enacted—in real-life, stressful situations—some of the key ingredients of empirically validated strategies for reducing problem drinking, specifically, identifying specific emotions and concomitant triggers related to stress and drinking as well as developing strategies for managing emotions and adhering to drinking-related goals. In future research, we will examine drinking outcomes over a longer duration to capture changes in alcohol consumption, frequency, and drinking patterns, including the contexts where participants utilize the sensorband and where they receive alerts. As academic or work issues constituted the vast majority of the issues participants chose when receiving an alert, we will be able to explore ways in which successfully managing stress by using the sensorband outside of drinking contexts assists students to reduce problem drinking.

We were particularly interested in understanding acceptability as measured by the amount of time participants spent on the app, particularly when responding to a sensor-triggered alert. Few studies of mHealth apps measure the amount of time participants spend on specific screens. Future research may entail examining how the amount of time spent on specific screens might mediate intervention effects, as well as understanding how contextual variables such as location or time of day effect the amount of time participants spend on their screen. We will examine patterns of screen time and how they relate to usability and outcomes with a larger sample. An important additional area of research entails specifying and individualizing the length of time individuals may need to use the technology in order to see changes in behavioral health patterns.

### Limitations

The major limitation of this pilot study is the very small sample size. Moreover, a limited number of sensorbands necessitated providing the sensorband to participants successively, restricting the amount of time participants’ could use the sensorband. Despite these limitations, results of this study demonstrate the potential to enhance mHealth interventions and help individuals adopt and sustain behavioral change. Delivering intervention material “just-in-time,” in the places and at the moment participants need to utilize behavioral change strategies learned in provider-delivered sessions has implications for deterring the drop-off typical of behavioral interventions and in particular, mHealth interventions [68]. Moreover, the results of this study have the potential to individualize treatment for patients in clinical settings where clinicians can review patients’ patterns of activities, and associated feelings and triggers, and discuss how these may change over time, relative to their environment, time of year, and developmental level, for example.

## Abbreviations

API: Application Programming Interface
AUDIT-C: Alcohol Use Disorder Identification Test-C
CBT: Cognitive Behavioral Therapy
CSQ: Client Satisfaction Questionnaire
EDA: Electrodermal activity
EMI: Ecological momentary interventions
GPS: Global Positioning System
MI: Motivational Interviewing
MtM: Mind the Moment
RA: Research Assistant
SECSAT: Southeastern Consortium for Substance Abuse Training
SNS: Sympathetic nervous system
TLFB: Time Line Follow Back
UHC: University health center
UTUAT: Unified Theory of Use and Acceptance of Technology
